# Inferior vena cava enteric fistula due to unresected colorectal metastasis

**DOI:** 10.1186/s13017-015-0024-7

**Published:** 2015-07-03

**Authors:** Hayim Gilshtein, Offir Ben-Ishay, Karina Nascovica, Yoram Kluger

**Affiliations:** Department of General Surgery, Rambam Health Care Campus, 8 Ha’Aliyah St, Haifa, 35254 Israel

## Abstract

A 57 year old male presented to our department with recurrent attacks of sepsis and upper gastrointestinal bleeding due to colorectal cancer metastasis that resulted in a fistula involving the inferior vena cava and the third part of the duodenum.

Four and a half years ago he underwent laparoscopic right hemicolectomy due to colonic adenocarcinoma.

A year prior to his recent hospitalization he underwent cytoreductive surgery followed by HIPEC due to peritoneal metastases in another hospital. During the operation a metastasis adherent to the inferior vena cava and the III part of the duodenum was revealed. The surgeon decided to mark the area with hemo- clips and after the patient recovered from surgery he was sent for radiotherapy aimed at controlling the left over metastases.

In his current hospitalization he underwent an en bloc resection of the III part of the duodenum, the adherent vena cava and the right kidney.

Gross pathology revealed a fistula between the vena cava and the duodenum with bile stained clot within the resected part of the vena cava.

The patient recovered well with resolutions of his presenting symptoms.

## Introduction

Colon cancer metastases are not rare. Liver, lung and peritoneal metastases are the most common sites [1]. Local recurrence, primarily in the site of a previous anastomosis is also well described [2]. The disease relapse pattern is related to both the cancer staging upon presentation and biologic characteristics of the tumor and host, only partially elucidated thus far.

The treatment of choice for resectable colon cancer is surgery. Adjuvant chemotherapy is tailored in line with the pathologic staging revealed after surgery. For local recurrent colon cancer, complete surgical excision, when applicable, remains the only option for cure. Surgery is also well proven for colorectal liver metastasis [3] with ensuing significant impact on disease free and 5-year survival. Moreover, in the last years surgery is offered in selected cases of colorectal peritoneal carcinomatosis in the form of cytoreductive surgery with heated chemotherapy (HIPEC) [4, 5].

Palliative resection of colorectal metastasis is usually limited to lesions causing significant bleeding, obstruction or perforation.

As it was described in the past [6] cytoreductive surgery with HIPEC might cause serious postsurgical complications such as anastomotic leaks, gastrointestinal bleeding and sepsis. Several other life threatening and rare complications were also reported. One of these is a case of colobronchial fistula [7].

Herein we describe a rare case of gastrointestinal bleeding and sepsis caused by a colon cancer metastasis involving the inferior vena cava, after cytoreductive surgery and HIPEC that was treated in our department.

## Case report

A 57-year old male presented to the emergency room with high grade fever and chills.

The patient was known to suffer from metastatic colon cancer. Following positive occult blood in his stool he was diagnosed in 2010 with colonic adenocarcinoma, involving the cecum, without any evidence of distant metastasis. A laparoscopic right hemicolectomy was performed. His final pathology revealed a T3N1 tumor. He received a FOLFOX adjuvant systemic chemotherapy.

The patient recovered well and proceeded with standard regular oncologic follow up including interval abdominal CT and PET scans as required.

About 3.5 years after the index operation several new lesions, suspicious of secondary spread were revealed. A lesion in the upper lobe of his right lung was resected thoracoscopically. Another lesion caused a significant obstruction of the right kidney that resulted in nephrostomy tube insertion.

The patient was offered a cytoreductive surgery with heated chemotherapy (HIPEC) with curative intent. At surgery HIPEC was performed as planned after resection and excision of all the abdominal load of metastases except for a solid lesion involving the IVC and the 3rd part of the duodenum, deemed unresectable. Due to its presumed irresectability the site was marked with metallic clips for later irradiation.

After surgery the patient underwent a targeted irradiation to the marked site and received additional course of systemic chemotherapy. He underwent further follow up with PET scans, revealing three main lesions with a high uptake on PET, at the previous anastomosis of the transverse colon with small bowel, in the omentum next to the anastomtic site and the metastsasis revealed at previous surgery involving the inferior vena cava, near the entrance of the right renal vein and the third part of the duodenum. There was no evidence of neither liver nor other distant metastasis.

The patient remained in a good general condition and performance status up to his presentation with fever and fatigue on 6 month after the second operation. His blood tests revealed anemia, high white blood count and CRP.

Blood cultures were taken and the patient was admitted for observation and empiric antibiotic treatment. During his long hospitalization in the internal medicine department his general condition continued to deteriorate with recurrent and persistent bouts of fever and sepsis. His blood cultures demonstrated a polibacterial flora with persistent candidemia as well. The flora of the microbiota suggested a GI tract source. Meanwhile, he received a wide spectrum antibiotic and antifungal therapy. During his stay, apart from fever he started to suffer from recurrent episodes of hematemesis and melena with symptomatic anemia that resulted in consumption of multiple blood products. He underwent a thorough workup for both his fever and suspected upper gastrointestinal bleeding. A chest and abdominal CT scans have not demonstrated disease progression, as compared to previous PET, with the same lesions shown, described previously. An upper endoscopy revealed blood clots in the stomach and first part of the duodenum and the metallic clips placed in the previous operation evidently penetrating inside the third portion of the duodenum. CT angioagraphy and a formal angiography failed to reveal an active source of bleeding, thus no angiographic intervention was performed.

**Fig. 1:**
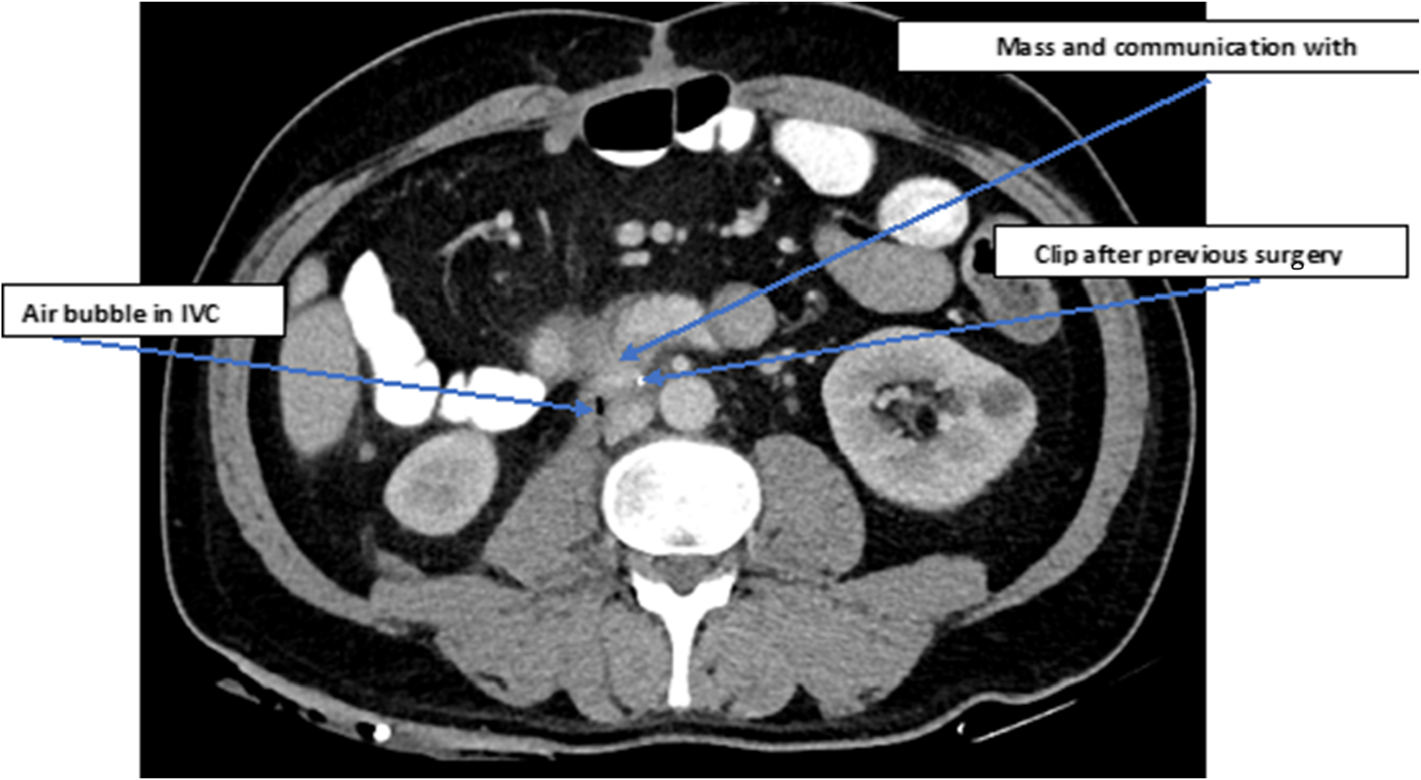
Abdominal CT. The mass shown with *arrows* indicating its connection to the IVC and clips from previous surgery

The patient was transferred to the department of general surgery for further observation and treatment. A revision of all the pertinent imaging modalities (Fig. 1) was performed and a multidisciplinary meeting was held to discuss the treatment options. A consensus was reached that the patient suffers from IVC-enteric fistula due to colon metastasis with an immediate life threatening potential and with a surgical salvage procedure being the only remaining viable option of treatment for palliation of his symptoms. The patient was informed and received a meticulous explanation of the planned procedure with its pure palliative intent. An informed consent was obtained.

At laparotomy a solid tumor lesion was found at the third and fourth parts of the duodenum. The inferior vena cava, at the level of the right renal vein appeared friable and almost necrotic, with a large opening in its anterior wall, covered by only a clot and fibrin, fistulazing to the third portion of the duodenum. En bloc resection of the tumors with the involved IVC, 3rd and 4th parts of the duodenum and right kidney (known as non-functional by DMSA and with previous nephrostomy) was completed.

The second part of the duodenum was anastomosed to the proximal jejunum. The tranverse colon anastomosis to the small bowel appeared intact. Another lesion in the omentum, measuring about 2 cm, at the proximity of the previous anastomosis, as shown on PET, was also excised. No reconstruction of the vena cava was performed, in consideration with avaibale collaterals. The surgery was completed with no evidence of gross disease apparent in the peritoneal cavity.

The final pathologic report revealed desmoplastic reaction and fibrosis, free of tumor in the main specimen and the omentum excised separately. Well differentiated colon adenocarcinoma with mucus production was found only in the margins of resected duodenum.

The patient underwent an uneventful recovery with return to good functional status, and resolution of both his fever and GI bleeding. Of notice, only minor peropheral edema developed after IVC resection.

## Discussion

Colorectal cancer metastasis remains a challenge. While surgery is well established for the treatment of liver metastasis, lung metastasis and also HIPEC gained acceptability in high volume centers for groups of patients with peritoneal carcinomatosis, there is still an ongoing debate for other types of metastasis. We presented a rare case of a symptomatic IVC-duodenal fistula treated successfully with surgical excision. It remains unclear what was the role of HIPEC and the later external beam irradiation on the development of this serious complication. The intent of the procedure was palliative and its influence on the patients’ overall survival is still questionable. However, we believe that for some, selected young patients a radical procedure might be undertaken prudently for either curative or palliative intent in cases of uncommon presentation of colorectal metastasis.

Dealing with complications of colorectal metastasis can be demanding with early involvement of experienced surgical team, in need of some specialized surgical procedures, as described herein.

## Consent statement

Written informed consent was obtained from the patient for publication of this Case report and any accompanying images. A copy of the written consent is available for review by the Editor-in-Chief of this journal.
